# A Case Report of Coronavirus Disease 2019 Presenting with Tremors and Gait Disturbance

**DOI:** 10.5811/cpcem.2020.5.48023

**Published:** 2020-05-20

**Authors:** Sharon Klein, Frederick Davis, Adam Berman, Shruti Koti, John D’Angelo, Nancy Kwon

**Affiliations:** * Donald and Barbara Zucker School of Medicine at Hofstra/Northwell, Department of Emergency Medicine, Hempstead, New York; † Long Island Jewish Medical Center, Department of Emergency Medicine, New Hyde Park, New York

**Keywords:** COVID-19, neurology, tremor

## Abstract

**Introduction:**

Neurologic symptoms present as significant complications of coronavirus disease 2019 (COVID-19) infection. This report describes a novel manifestation of tremors triggered by severe acute respiratory syndrome coronavirus 2 infection.

**Case Presentation:**

We describe a case of a 46-year-old man with COVID-19 infection complicated by a bilateral intention tremor and wide-based gait. Although neurological manifestations have been reported related to COVID-19, tremulousness has not yet been described.

**Conclusion:**

Considering the evolving diversity of neurologic manifestations in this infection, emergency physicians should be vigilant of possible COVID-19 infection in patients presenting with unexplained neurologic symptoms.

## INTRODUCTION

Coronavirus disease 2019 (COVID-19) was first reported in December 2019, originating from Wuhan, China, as an aggressive viral pneumonia with poorly understood pathophysiology. As the caseload has grown exponentially across the United States, we are seeing a variety of clinical presentations affecting a multitude of organ systems. Emergency providers need to be able to recognize these presentations as possible sequelae of COVID-19 infection to triage and isolate patients during evaluation. Neurologic symptoms present as significant complications of COVID-19 infection. This report describes a novel manifestation of tremors triggered by severe acute respiratory syndrome coronavirus 2 (SARS-CoV-2) infection.

## CASE REPORT

A 46-year-old male was brought to the emergency department (ED) with complaints of two weeks of cough, fever, generalized myalgias, sore throat, with progressively worsening of shortness of breath, and night sweats. He was initially treated with amoxicillin-clavulanate for pneumonia for seven days as prescribed by his primary care physician. On day eight he began to have tremors without fevers, which resulted in difficulty ambulating. He denied any nausea, vomiting, diarrhea, constipation, chest or abdominal pain. He had no other relevant medical history, denied taking any other medications, and denied history of alcohol use. Before going into self-quarantine he noted that some of his co-workers were having flu-like symptoms but he was unaware whether they had been tested for COVID-19.

On physical examination in the ED his vital signs were blood pressure 130/87 millimeters of mercury, temperature 36.6° Celsius (97.9° Fahrenheit), pulse rate 108 beats per minute, respiratory rate 22 breaths per minute, and oxygenating at 96% on room air. On respiratory exam, he had clear and equal breath sounds bilaterally. Neurologic exam revealed intact mental status that was oriented to self, date, and place. He had no dysarthria, aphasia, or neglect. His cranial nerves exam was significant for saccadic intrusions with smooth pursuit. A generalized tremor was noted when the patient was lying down, which worsened with movement, and there was a postural tremor in all extremities. Heel-to-shin exam was non-dystaxic although tremulous, and there was a bilateral intention tremor. On motor exam, he had normal tone and five out of five strength of all muscle groups in the upper and lower extremities. He was noted to have a wide-based gait with unsteadiness, but there was no dysmetria, pronator drift or truncal ataxia. His sensation was intact to light touch. No other abnormalities were noted on physical exam.

In the ED he was evaluated by neurology due to the constant tremors. Computed tomography (CT) of the head and CT angiogram did not reveal any significant findings, toxicology report came back negative, and thyroid-stimulating hormone, thiamine, and folate levels were normal. Chest radiograph showed clear lungs without any focal consolidation. Magnetic resonance imaging (MRI) done during his hospital stay showed hyperintense foci in the bifrontal subcortical and deep white matter on scattered T2-weighted, fluid-attenuated inversion recovery. These findings likely represent sequalae of microangiopathic ischemic changes. His hospital course was uncomplicated, and respiratory status improved with supportive measures. Final impression by neurology was that these were essential tremors, and the decision was made to treat with propranolol from which patient reported some mild improvement of symptoms.

## DISCUSSION

Virology studies of SARS-CoV-2 and Middle Eastern respiratory syndrome coronavirus (MERS-CoV) have shown their ability to enter the brain and spread to specific areas such as the thalamus and brainstem, although the route of entry has yet to be elucidated.^1^ Given this, it is likely that SARS-CoV-2 has similar neuro-invasive potential.^1^ Multiple neurologic manifestations have been reported among patients hospitalized with COVID-19. In a case series of 214 patients with COVID-19 in Wuhan, China, neurological symptoms were present in 36.4% of patients, particularly with a preference for those with more severe infection as according to their respiratory status. The most common nervous system complications were dizziness and headache among those with central nervous system manifestations, and taste and smell impairment in those with peripheral nervous system impairment.^2^ This case to our knowledge is the first case of tremors described in the COVID-19 pandemic. Similar neurologic manifestations, with postural and action tremors, have been reported with other viral infections. A case study involving a hepatitis C virus-positive patient reported these isolated symptoms despite normal MRI findings.^3^ In pediatric patients gait unsteadiness has been attributed to acute cerebellar ataxia secondary to numerous viral infections ranging from varicella to coxsackievirus.^4^

**CPC-EM Capsule**

What do we already know about this clinical entity?

Coronavirus disease 2019 (COVID-19) typically presents with symptoms of fever, cough, fatigue, and myalgias, but can rapidly progress to involve other organ systems.

What makes this presentation of disease reportable?

Although postural and action tremors have been seen with other viral infections, this is the first known presentation linking these symptoms with COVID-19.

What is the major learning point?

COVID-19 can present with various neurologic manifestations such as headache, dizziness, taste and smell impairments, ataxia, seizures and tremors.

How might this improve emergency medicine practice?

Unexplained tremors and gait abnormalities can be a rare presentation of COVID-19 infection, and should be suspected in patients presenting with viral syndrome.

## CONCLUSION

Considering the prevalence of neurologic manifestations occurring in this illness, physicians should consider SARS CoV-2 infection in patients presenting with unexplained neurologic symptoms to avoid delayed diagnosis and prevention of transmission.
